# O028. Thalamo-cortical network changes during the migraine cycle: insights from MRI-based microstructural and functional resting-state network correlation analysis

**DOI:** 10.1186/1129-2377-16-S1-A52

**Published:** 2015-09-28

**Authors:** Gianluca Coppola, Antonio Di Renzo, Emanuele Tinelli, Chiara Lepre, Elisa Iacovelli, Cherubino Di Lorenzo, Giorgio Di Lorenzo, Vincenzo Parisi, Mariano Serrao, Flavia Pauri, Giancarlo Fiermonte, Claudio Colonnese, Jean Schoenen, Francesco Pierelli

**Affiliations:** 1 Department of Neurophysiology of Vision and Neuro-ophthalmology, G.B. Bietti Foundation IRCCS, Rome, Italy; 2 Department of Neurology and Psychiatry, Neuroradiology section, “Sapienza” University of Rome, Rome, Italy; 3 Department of medico-surgical sciences and biotechnologies, Neurology section, “Sapienza” University of Rome, Rome, Italy; 4 Don Carlo Gnocchi Onlus Foundation, Milan, Italy; 5 Department of Systems Medicine, University of Rome “Tor Vergata”, Laboratory of Psychophysiology, Psychiatric Clinic, Rome, Italy; 6 Department of medico-surgical sciences and biotechnologies, “Sapienza” University of Rome Polo Pontino, Latina, Italy; 7 Headache Research Unit, Department of Neurology-CHR Citadelle, University of Liège, Belgium; 8 IRCCS Neuromed, Pozzilli, IS Italy

## Background

Abnormal structural and functional plasticity in cortical and subcortical brain regions may be an important aspect of migraine pathophysiology. Resting state magnetic resonance imaging allows studying functionally interconnected brain networks. Whether there is a relation between the plasticity of resting state networks and integrity of thalamic microstructure during the migraine cycle is not known. To verify functional connectivity between brain networks at rest and its relationship with thalamic microstructure in migraine without aura (MO) patients during and between attacks.

## Methods

Twenty-four patients with untreated MO underwent 3T MRI scans during (n=10) or between attacks (n=14) and were compared to a group of 15 healthy volunteers. We used MRI to collect resting state data among four selected resting state networks, identified using group independent component (IC) analysis. Fractional anisotropy (FA) values of bilateral thalami were retrieved from a previous diffusion tensor imaging study on the same group of subjects and correlated with resting state ICs Z-scores.

## Results

We found a significant reduced functional connectivity between the default mode network and the visuo-spatial system between attacks, and between the executive control network and the dorso-ventral attention system during attacks. When HV and migraine groups were combined, ictal and interictal selected ICs Z-scores correlated negatively with bilateral thalami FA values.

## Conclusions

The present results are the first evidence supporting the hypothesis that abnormal dynamics of the connectivity between thalamus and functional cerebral networks at rest could contribute to the recurrence of migraine attacks.

Written informed consent to publication was obtained from the patient(s).

